# Expanded porphyrins: functional photoacoustic imaging agents that operate in the NIR-II region†

**DOI:** 10.1039/d1sc01591e

**Published:** 2021-06-23

**Authors:** Jingqin Chen, Adam C. Sedgwick, Sajal Sen, Yaguang Ren, Qinchao Sun, Calvin Chau, Jonathan F. Arambula, Tridib Sarma, Liang Song, Jonathan L. Sessler, Chengbo Liu

**Affiliations:** Research Center for Biomedical Optics and Molecular Imaging, Shenzhen Institute of Advanced Technology, CAS Key Laboratory of Health Informatics, Chinese Academy of Sciences Shenzhen 518055 China cb.liu@siat.ac.cn; Department of Chemistry, University of Texas at Austin 105 East 24th Street A5300 Austin Texas 78712-1224 USA kumartridib@gmail.com sessler@cm.utexas.edu

## Abstract

Photoacoustic imaging (PAI) relies on the use of contrast agents with high molar absorptivity in the NIR-I/NIR-II region. Expanded porphyrins, synthetic analogues of natural tetrapyrrolic pigments (*e.g.* heme and chlorophyll), constitute as potentially attractive platforms due to their NIR-II absorptivity and their ability to respond to stimuli. Here, we evaluate two expanded porphyrins, naphthorosarin (**1**) and octaphyrin (**4**), as stimuli responsive PA contrast agents for functional PAI. Both undergo proton-coupled electron transfer to produce species that absorb well in the NIR-II region. Octaphyrin (**4**) was successfully encapsulated into 1,2-distearoyl-*sn*-glycero-3-phosphoethanolamine-poly(ethylene glycol) (DSPE-PEG_2000_) nanoparticles to afford **OctaNPs**. In combination with PAI, **OctaNPs** allowed changes in the acidic environment of the stomach to be visualized and cancerous *versus* healthy tissues to be discriminated.

## Abstract 2

In this study, two expanded porphyrins, octaphyrin and naphthorosarin were evaluated as potential PA agents. The nanoparticle encapsulation of octaphyrin successfully enabled the visualization of acidic environments and the discrimination between cancerous and healthy tissues.

## Graphical abstract

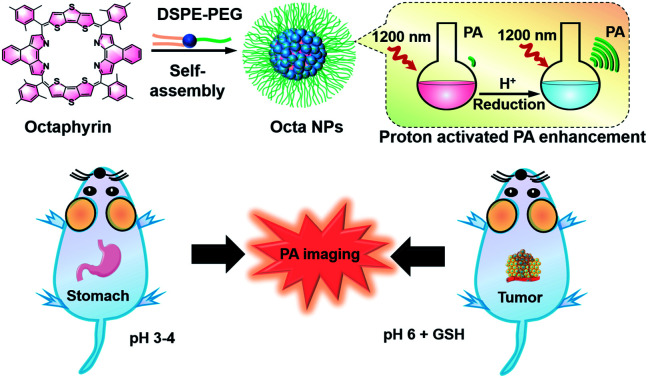

## 1. Introduction

**Fig. 1:**
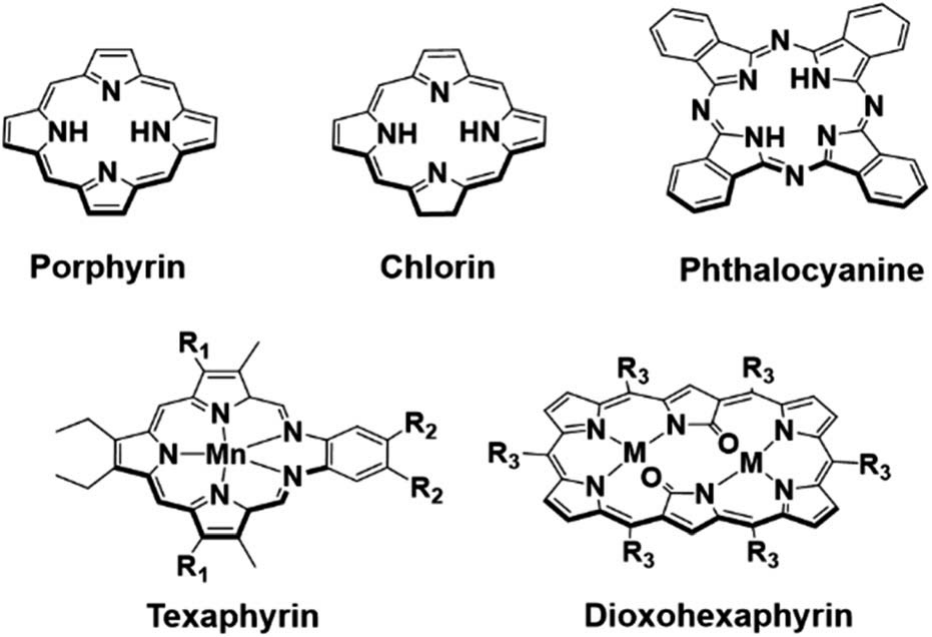
Top row: chemical structures of the porphyrin, chlorin and phthalocyanine scaffolds. Bottom row: chemical structures of manganese texaphyrin (R_1_ = –CH_2_CH_2_CH_2_OH, R_2_ = –(CH_2_CH_2_O)_3_CH_3_) and 26 π-electron-conjugated bis-metal (Zn and Cu) dioxohexaphyrin complexes (M = Cu or Zn, R_3_ = C_6_F_5_) reported by Furuta and collaborators.^22^

Photoacoustic imaging (PAI) is an imaging modality that combines the high contrast and sensitivity of optical imaging with the tissue penetration depths of ultrasound (US).^1^ This “light in – sound out” approach relies on the light absorption of either an endogenous or exogenous chromophore, typically excited by a pulsed laser, to produce heat and generate acoustic pressure waves (thermoelastic expansion).^2,3^ These acoustic signals are then detected using ultrasound transducers and reconstructed to form the photoacoustic (PA) image. One of the most important preclinical and clinical applications of PAI is mapping blood oxygenation within tissue through the excitation of the endogenous chromophore, hemoglobin, (Hb).^1,4–6^ Heme is an iron porphyrin (*cf.*Fig. 1 for generic structure) that displays distinct absorption differences between its unbound and oxygen-bound forms, allowing imaging of both oxygenated and deoxygenated tissues.^4^

In recent years, a number of classic tetrapyrrolic pigments, including porphyrins, chlorins and phthalocyanines, have been demonstrated as PA-responsive systems for the detection of biologically important species (*i.e.* low pH, enzymes, and hydrogen peroxide (H_2_O_2_)).^7–10^ However, most tetrapyrrolic PA systems are limited to the NIR region (NIR-I: 700–950 nm).^7,11^ Absorption greater than 1000 nm, termed the second near-infrared region (NIR-II: 1000–1350 nm), allows for deeper tissue penetration and reduced light scattering.^12–16^ Current NIR-II PA contrast agents comprise of organic semiconducting conjugated polymers; very few are solely organic-based.^17,18^ In recent years, numerous groups have focused on developing new porphyrinoid systems.^19,20^ Many of these, particularly the so-called expanded porphyrins, show promise as NIR-I and NIR-II absorbers.^20–23^ Early on, our group reported a penta-aza Schiff base porphyrinoid known as texaphyrin that absorbs >700 nm and forms stable 1 : 1 complexes with a large array of metal cations. Recently, a Mn(ii) texaphyrin was shown to be effective as a PA contrast agent using NIR-I light (Fig. 1).^24^ To broaden the application of PAI, it is useful to develop systems that are capable of absorbing in the NIR-II region. In 2020, Furuta and collaborators reported 26 π-electron-conjugated bis-metal (Zn and Cu) dioxohexaphyrin complexes as potential NIR-II PA contrast agents (Fig. 1).^25^ Since then, Furuta and collaborators have reported several other expanded porphyrin platforms that produce a PA signal in the NIR-II and NIR-III region (NIR-III: 1550–1870 nm).^15,26–28^ However, no biological studies were carried out nor were these compounds shown responsive to biological stimuli. There thus remains an unmet need for functional PAI agents that function in the NIR-II region. Here, we report the use of two expanded porphyrins, naphthorosarin (**1**) and octaphyrin (**4**), as proton-coupled electron transfer (PCET)-activated PA imaging agents for functional PAI.^29,30^ As detailed below, octaphyrin (**4**) proved effective for the imaging of stomach pH and allowed the discrimination between cancerous tissue (HepG2) and healthy tissue in BALB/c nude mice model.

## 2. Results and discussion

**Scheme 1:**
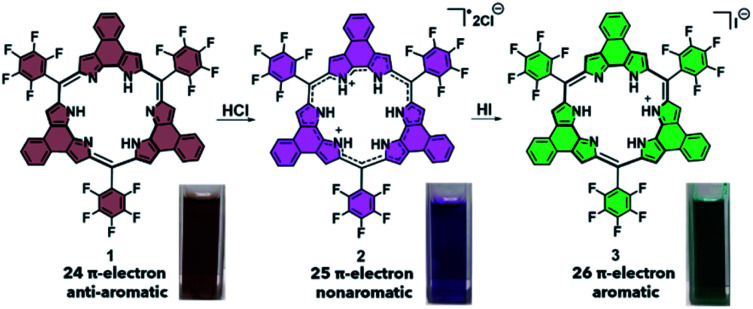
PCET-mediated reduction of naphthorosarin **1** using HCl and HI and photographs of each species in dichloromethane (DCM).

**Scheme 2:**
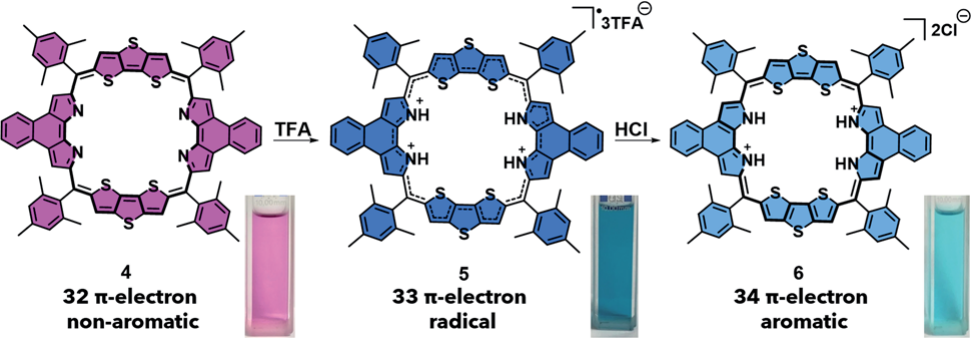
PCET-mediated reduction of octaphyrin **4** using TFA and HCl and the photographs of each species in DCM.

The two porphyrinoids considered in this study are naphthorosarin **1** and octaphyrin **4** (Schemes 1 and 2).^29,30^ Unique to both systems is their propensity to undergo PCET, this process is currently being used to describe any reaction that involves both a proton transfer (PT) and electron transfer (ET).^31–33^ In the case of naphthorosarin **1** and octaphyrin **4**, it is believed that both undergo stepwise proton transfer electron transfer (PTET) or concerted proton electron transfer (CPET) processes depending on the chosen acid and reductant.^29,30^ The addition of a proton source and an oxidizable anion, *e.g.*, HCl, to 24 π-electron antiaromatic **1** results in a quasi-stable non-aromatic triprotonated monoradical dication 25 π-electron species (**2**) being readily formed.

This radical is characterized by an absorption wavelength at ∼900 nm. In the presence of stronger reductants, such as HI, conversion to the two-electron reduced 26 π-electron aromatic species (**3**), absorbing at *ca.* 1000 nm, occurs (Scheme 1). In contrast, the 32 π-electron non-aromatic octaphyrin **4** undergoes a concerted two-electron reduction in the presence of a proton source such as HCl. This yields the corresponding 34 π-electron aromatic form (**6**) with an absorption maximum at *ca.* 1200 nm (Scheme 2). In the presence of less redox active acid trifluoroacetic acid (TFA), a 33 π-electron radical **5** is formed, which can then be further reduced with the addition of reductants.^30^ Therefore, a critical feature to PCET is that it requires both a reductant and a proton source. To our knowledge this has not previously been explored in the context of photoacoustic imaging.

The importance of pH in human health is well appreciated. For instance, numerous studies have shown that changes in the upper gastrointestinal tract pH are implicated in pathological processes.^34,35^ A high pH has been observed in the stomach of gastric ulcer (pH = 3.4) and gastric cancer patients (pH = 6.6) compared to healthy subjects (pH = 2.9), whereas, a low pH has been seen in the stomach of esophageal ulcer (pH = 1.9) and duodenal ulcer patients (pH = 2.1).^35^ Thus, the ability to probe non-invasively the dynamics of stomach pH could prove useful in monitoring stomach health. Cancer, broadly speaking represents another area where monitoring pH *via* PAI could be beneficial in the context of diagnostic and therapeutic applications.^8,36^ It is well-known that the extracellular tumor microenvironment is slightly acidic, pH = 6.4–7.0.^37^ Most cancer environments are also highly reducing.^37,38^ We thus postulated that stomach and cancer imaging would provide useful testbeds for evaluating **1** and **4** as possible PCET-based PAI agents.

The pH responsiveness of **1** and **4** were evaluated in THF solution through the careful addition of 1 M aqueous HCl. Both **1** and **4** demonstrated a strong PA response when subject to 900 and 1200 nm pulsed laser excitation (15 mJ), respectively, as the apparent^39^ pH decreased (see Fig. S1–S3†). This was considered indicative of the formation of the non-aromatic triprotonated monoradical dication 25 π-electron species (**2**) and 34 π-electron aromatic (**6**), respectively. No 26 π-electron naphthorosarin species (**3**) was observed, presumably reflecting its less positive reduction potential ((**1**: +0.42 V and +0.04 V); (**4**: +0.56 V and +0.25 V)).^25,26^

**Fig. 2:**
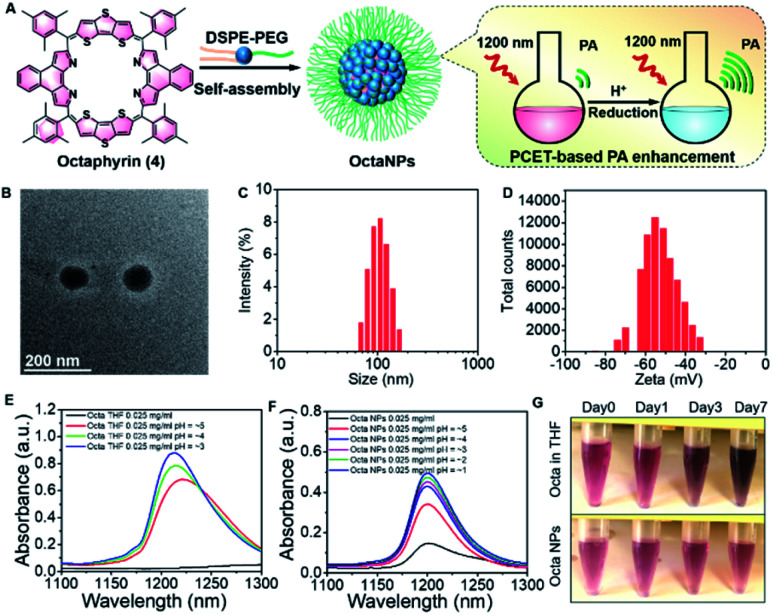
(A) Preparation of nanoparticles (**OctaNPs**) from octaphyrin **4**. (B) TEM image of **OctaNPs**. (C and D) Size and zeta potential distribu-tion of **OctaNPs**. (E and F) Absorbance spectra of **4** in THF at varying apparent^39^ pH values and **OctaNPs** in aqueous solution at varying pH values. pH was adjusted through the careful addition of 1 M HCl. (G) Stability studies of octaphyrin **4** in THF and **OctaNPs** in neutral aqueous media (pH = 7.40) that involved monitoring over the course of 7 days at room temperature.

A nanoparticle (NP) encapsulation strategy was employed to allow studies of **1** and **4** in biological milieus. Both **1** and **4** could be encapsulated using 1,2-distearoyl-*sn*-glycero-3-phosphoethanolamine-poly(ethylene glycol) (DSPE-PEG_2000_) (compound: DSPE-PEG_2000_ = 1 : 750 (w/w)). Unfortunately, the absorption and PA features of **NaphthNPs** (from **1**) proved unresponsive to pH changes (see ESI – Fig. S4 and S5†). In the case of the **OctaNPs** (from **4**) the mean size (106 nm) and zeta potential (−55 mV) were considered suitable for biological testing (Fig. 2D). The ratio between **4** and DSPE-PEG_2000_ was found to be critical for the PCET-mediated conversion of **4** to **6** (see ESI – Fig. S6†). No changes in acidic media were observed for **OctaNPs** at ratios 1 : 10 and 1 : 100 (**4**:DSPE-PEG_2000_). On the other hand a ratio of 1 : 750 (**4**:DSPE-PEG_2000_) was found to provide an optimal formulation for the formation of **6** in acidic media. A similarly responsive formulation was not found in the case of the **NaphthNPs**. While studies are ongoing in an effort to determine an optimal nanoparticle strategy for **1**, this failure is ascribed to the less positive reduction potential of this particular expanded porphyrin. No changes in color were observed for **OctaNPs** in aqueous solution over the course of 7 days. In contrast, **4** in THF was observed to gradually became darker in color, which we ascribe to partial degradation (Fig. 2G). On this basis, we suggest that the use of this nanoparticle strategy improves the solution phase stability of **4**. Moreover, **OctaNPs** displayed a pH dependent increase in the long-wavelength absorption band expected for a PCET process wherein the components of the medium (*e.g.*, chloride anions) serve as the reductant (Fig. 2E and F). The importance of reductants to this PCET-response was further confirmed using the less-redox active acid trifluoroacetic acid (TFA) to adjust the pH of the aqueous test solutions. At pH 5, a minimal increase in absorption at ∼1200 nm was observed for **OctaNPs**. In contrast, co-treatment with both TFA and the biological reductant glutathione (GSH, 40 μM) led to a significant increase in this absorption feature (Fig. S7†). We thus focused our attention on **OctaNPs** for the remainder of this study.

**Fig. 3:**
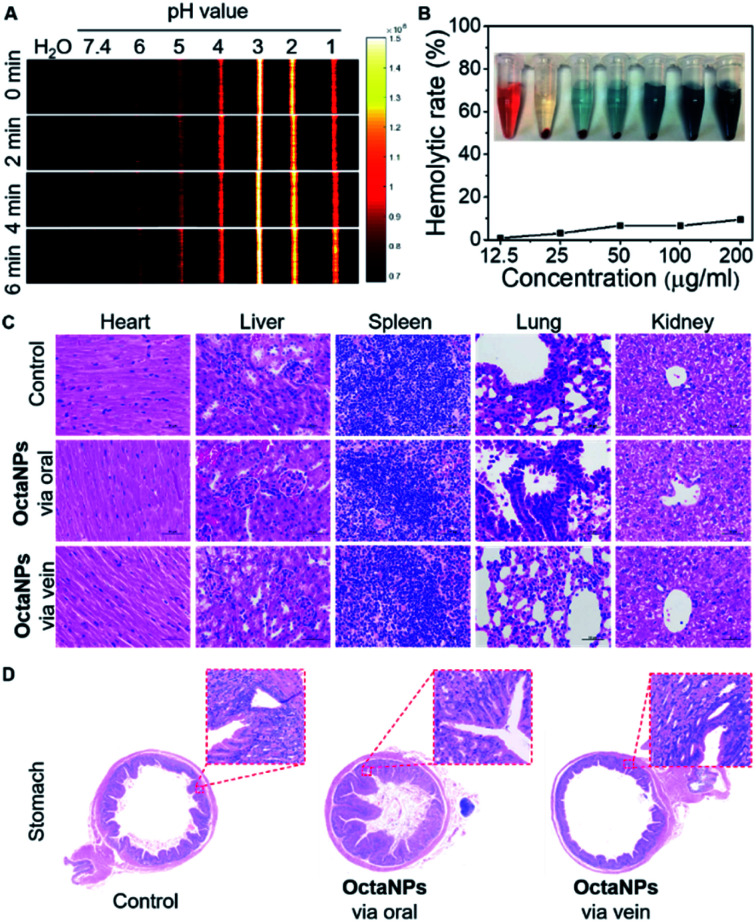
(A) Photoacoustic images (PAI) of aqueous **OctaNPs** solutions (0.1 mg mL^−1^) across various pH values recorded at different time points. (B and C) Hemolytic rate of **OctaNPs** and (C and D) HE staining images of the major organs (including heart, liver, spleen, lung, kidney) and stomach from mice treated with neutral aqueous solutions of **OctaNPs** (5 mg kg^−1^) through oral and tail vein injection for 7 days, respectively. Magnification × 400.

Next, the ability of **OctaNPs** to detect changes in pH was evaluated using PAI. The maximum PA intensity was seen at pH = 3 with the response being essentially immediate on the laboratory time scale. In contrast, a gradual turn-on response was observed over the 5–6 pH range that peaked after 6 min (Fig. 3). The lower PA signals observed at pH 1 and pH 2 are ascribed to partial degradation of **4**. No adverse toxicities were seen for **OctaNPs**, as inferred from histological analyses and evaluation of hemolysis rates (Fig. 3B and C).

**Fig. 4:**
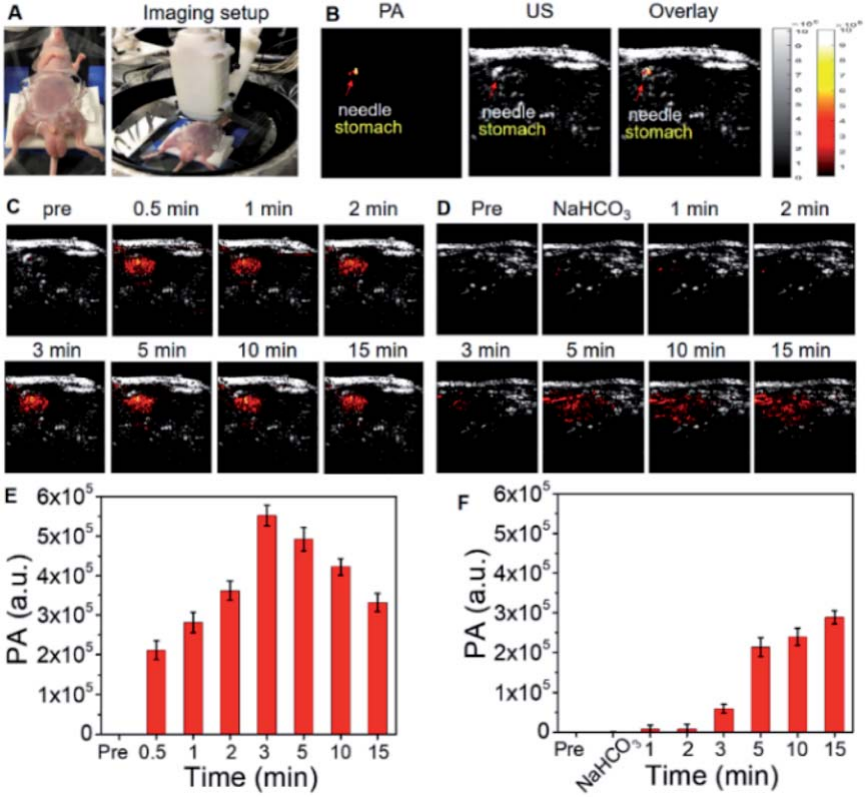
(A) Photographs of the PA imaged mouse and the setup of the PAI probe. (B) PA and ultrasound (US) B-scan images of the stomach area, into which the administration needle is inserted. (C) PA and US B-scan images of the stomach area before and after injection with **OctaNPs** (200 μL, 0.1 mg mL^−1^, neutral aqueous media) under 1200 nm pulse laser excitation. (D) PA and US B-scan images of stomach area before, pre-treatment with saturated NaHCO_3_ (50 μL), and after injection with **OctaNPs** (200 μL, 0.125 mg mL^−1^) under 1200 nm pulse laser excitation. (E and F) Quantified PA signal corresponding to (C and D). Sample size: *n* = 3.

**OctaNPs** were then tested as PAI agents *in vivo*. As shown in Fig. 4, direct injection (intragastric) of **OctaNPs** (200 μL, 0.1 mg mL^−1^) into the stomach resulted in an immediate PA signal being observed (Fig. 4C and E). This is due to the presence of HCl within the stomach and the stomach having a known pH range of 1.5–3.5. These environment are thus conducive to PCET reduction of **4**.^34^ To test whether **OctaNPs** could be used not just to visualize the stomach, but to monitor directly dynamic changes in the stomach pH, mice were pretreated through the injection of a saturated NaHCO_3_ solution (50 μL) to raise the pH (Fig. 4D and F). This was followed by the direct injection of **OctaNPs** (200 μL, 0.1 mg mL^−1^, neutral aqueous) into the stomach. No PA signal was initially observed under 1200 nm excitation (15 mJ), a finding ascribed to the bicarbonate-induced neutralization of the gastric acid. As time progressed the PA signal at 1200 nm increased reflecting the presumed secretion of gastric acid by the mice.

**Fig. 5:**
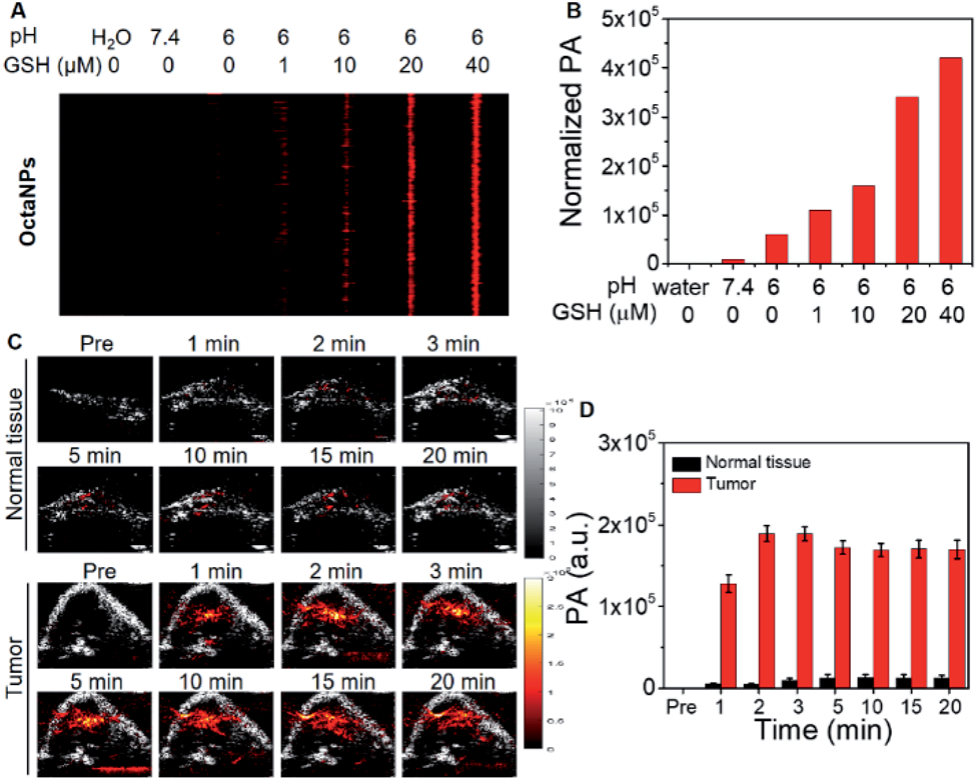
(A and B) PA images and quantified PA signals of **OctaNPs** in an aqueous pH 6 medium with increasing concentrations of GSH (0–40 μM). (C) PA and US B-scan images of normal tissue (right flank of mouse) and tumor before and after *in situ* injection with **OctaNPs** (200 μL, 0.1 mg mL^−1^) under 1200 nm pulse laser excitation. (D) Quantified PA signal in normal tissue and in a HepG2 mice model. Sample size: *n* = 3.

Efforts were then made to explore **OctaNPs** as PCET-triggered PA agents for cancer imaging. As noted above, the extracellular tumor microenvironment is slightly acidic (pH 6.4–7.0).^37^ As shown in Fig. 3A, **OctaNPs** produce a minimal PA “turn on” response at 1200 nm at pH 6. However, we considered it likely that the reducing nature of tumor tissues^38,40^ would facilitate PCET and afford a significant PA response at 1200 nm. To test this hypothesis, increasing concentrations of the endogenous reducing agent GSH were added to a pH 6 solution containing **OctaNPs**. This resulted in a marked increase in the PA signal intensity (Fig. 5A and B). In contrast, no changes in absorptivity were observed when GSH was added to a neutral pH 7.4 solution of **OctaNPs** (see ESI – Fig. S8†). **OctaNPs** were then injected into the right flank of BALB/c nude mice as well as into a HepG2 mice tumor model. A statistically significant PA signal at 1200 nm was observed for the tumor region into which the **OctaNPs** (200 μL, 0.1 mg mL^−1^) had been injected. In contrast, minimal PA signals were observed for healthy tissues. Overall, 2 minutes post-injection, a 42 (±7)-fold difference in PA signal intensity between cancerous and healthy tissue was observed (Fig. 5C and D).

## 3. Conclusion

The results presented here lend support to the suggestion that expanded porphyrins could prove useful as PAI agents. Systems such as **4**, that are known to undergo PCET, provide for environmental responsive PA imaging, while allowing access to the NIR-II spectral region. Encapsulation of **4** in DSPE-PEG afforded biocompatible nanoparticles (**OctaNPs**) that were shown to be stable for over 7 days. **OctaNPs** enabled the visualization of acidic environments such as in the stomach, along with changes in the stomach pH. **OctaNPs** also proved effective at discriminating between cancerous and healthy tissues with a 42-fold difference in the PA intensity being observed. Overall, this work serves to highlight the role that expanded porphyrins may have to play in functional photoacoustic imaging in the NIR-II region.

## Ethical statement

All animal experiments and procedures were performed in compliance with the requirements of the National Act on the Use of Experimental Animals (People's Republic of China) and were approved by the Experimental Animal Ethical Committee of Shenzhen Institute of Advanced Technology, Chinese Academy of Sciences. The accreditation number is SIAT-IRB-180205-YYS-CJQ-A0413.

## Data availability

All relevant data supporting the key findings of this study are available within the article and its ESI or from the corresponding author upon request.

## Author contributions

J. Chen and A. C. Sedgwick conceived the project and designed the experiments. J. Chen performed the nanoparticles preparation, characterization and biological experiment. A. C. Sedgwick and S. Sen synthesized compounds. A. C. Sedgwick and J. Chen wrote the paper and prepared the manuscript. Y. Ren conducted partial PA imaging experiments. J. L. Sessler and C. Liu contributed writing, funding acquisition and supervision. All authors provided input on the manuscript.

## Conflicts of interest

J. F. A. and J. L. S. currently serve, respectively, as Vice President and a non-executive Director for OncoTEX, Inc. that provided partial support for this work.

## Supplementary Material

SC-012-D1SC01591E-s001
