# Supplementary material for: Expanded porphyrins: functional photoacoustic imaging agents that operate in the NIR-II region
Source: Chem Sci. 2021 Jun 23;12(29):9916–21. doi: 10.1039/d1sc01591e (PMC8317656; doi:10.1039/d1sc01591e)
Supplement: SC-012-D1SC01591E-s001 [file SC-012-D1SC01591E-s001.pdf]

Electronic Supplementary Material (ESI) for Chemical Science.
This journal is © The Royal Society of Chemistry 2021

Expanded porphyrins: Functional photoacoustic imaging agents that operate in the NIR-II region

Jingqin Chen,[1,a] Adam C. Sedgwick,[2,a] Sajal Sen,[2,a] Yaguang Ren,[1] Qinchao Sun,[1] Calvin Chau,[2] Jonathan F. Arambula,[2] Tridib Sarma,[2,*] Liang Song,[1] Jonathan L. Sessler,[2*] and Chengbo Liu[1*]

[1] Research Center for Biomedical Optics and Molecular Imaging, Shenzhen Institute of Advanced Technology, CAS Key Laboratory of Health Informatics, Chinese Academy of Sciences, Shenzhen 518055, China.

[2]Department of Chemistry. University of Texas at Austin, 105 East 24th Street A5300, Austin, Texas 78712-1224, United States.

[a]Equal contribution

*Corresponding authors and emails:

Prof. Jonathan L. Sessler: sessler@cm.utexas.edu

Prof. Chengbo Liu: cb.liu@siat.ac.cn

Dr. Tridib Sarma: kumartridib@gmail.com

Table of Contents

1. Experimental and General Methods S3

2. Additional Figures S6

3. References S9

1. Experimental and General methods

1.1 Materials.

DSPE-PEG$_{2000}$ was purchased from Xi'an Ruixi Biological Technology Co., Ltd (Xi'an, China). Dulbecco's Modified Eagle's Medium (DMEM), penicillin–streptomycin solution, fetal bovine serum (FBS), trypsin-EDTA solution and phosphate buffer solution (PBS) were purchased from Gibco (AG, USA). All other chemicals were bought from Aladdin (Shanghai, China).

1.2 Octaphyrin and Naphthorosarin synthesis

Naphthorosarin (**1**) and octaphyrin (**4**) were synthesized and isolated as previously reported.[1-2]

1.3 OctaNPs and NaphthNPs: Synthesis and characterization

1 mg of **1** or **4** and 750 mg of DSPE-PEG$_{2000}$ were dissolved in 5 mL of tetrahydrofuran (THF) and then sonicated for 5 min. The volatiles were evaporated off at room temperature. Deionized water (4 mL) was then added to the resulting mixture, which was sonicated for 10 min. This afforded solutions of **OctaNPs** and **NaphthNPs**. The UV-Vis-NIR absorption spectra were recorded on a PerkinElmer Lambda 950 spectrophotometer. The morphology of the nanoparticle was evaluated using a transmission electron microscope (TEM, FEI Tecnai G2 F20 S-Twin). The size of the nanoparticles was determined via dynamic laser scattering (DLS, Zetasizer Nano-ZS, Malvern Instruments, UK).

1.4 PA equipment.

A photoacoustic computed tomography (PACT) system was used for this work. An optical parametric oscillator (OPO) laser source (Innolas GmbH, Bonn, Germany) emitting 8 ns pulsed lasers was coupled to a multimode optical fiber with a 1500 um core diameter for the photoacoustic signal excitation. The use of this nanosecond pulsed laser on the tumor tissue resulted in very small thermal expansions. This led to the vibration of the tissue, which generated ultrasonic waves that could be recorded by a commercial 128-element

linear array transrectal ultrasound transducer. The Q-switch output of the laser source was synchronized with a Vantage 128 research ultrasound platform (Verasonics, Inc. Kirkland WA, USA) to allow photoacoustic and ultrasound data acquisition. Photoacoustic signals were acquired at a frame rate equal to the laser pulse firing frequency of 30 Hz, and a conventional delay and sum (DAS) reconstruction algorithm was used to reconstruct the photoacoustic B-scan image. The system can simultaneously display both photoacoustic and ultrasound images in real-time.

1.5 Cell line and animal studies

All animal handling and experimental procedures were performed in compliance with the Animal Study Committee of the Shenzhen Institutes of Advanced Technology, Chinese Academy of Sciences. The human liver cancer cell line (Hep G2) was obtained from the American Type Culture Collection (ATCC). HepG2 cells were cultured in DMEM medium supplemented with 10% (v/v) fetal bovine serum, 1% (v/v) penicillin or 1% (v/v) streptomycin and incubated at 37 °C with 5% CO_2 atmosphere. Male BALB/c nude mice (5-8 weeks old) were obtained from the Medical Experimental Animal Center of Guangdong Province (Guangzhou, China) and used for this work. The HepG2 tumor model was established by subcutaneous injection of 5×10^6 cells (in 150 μL PBS) into the back of BALB/c nude mice.

1.6 *In vitro* hemolysis test

Blood from BALB/c nude mice was collected, centrifuged (2500 rpm, 3 min), and washed 3 times with PBS to separate the red blood cells (RBC) from the plasma. 10% RBC (v/v) in PBS was incubated with different concentrations of nanoparticles (12.5, 25, 50, 100, and 200 μg/mL) for 1 h at 37 °C. The mixture was then centrifuged (2500 rpm, 1 min) and the supernatant of the suspensions was collected and analyzed by an UV-Vis-NIR spectrometer at 541 nm wavelength. The hemolytic ratio (HR) was calculated using the following equation:

$$\mathrm{HR}\ (\%) = \frac{\mathrm{OD}_t - \mathrm{OD}_{nc}}{\mathrm{OD}_{pc} - \mathrm{OD}_{nc}} \times 100\%$$

Where OD_t, OD_{pc} and OD_{nc} are the absorbance of the tested samples, positive (deionized water) and negative (PBS) controls, respectively.

1.7 *In vivo* PAI

Mice (n = 3) were kept under anesthesia using 1.5% isoflurane mixed with pure oxygen and the body temperature was maintained at 37.5 °C by using a heating pad. For stomach imaging, photoacoustic images of the stomach regions were obtained before and after intragastric administration of the **OctaNPs** (0.1 mg/mL, 200 μl). For tumor imaging, photoacoustic images of tumor regions were obtained before and after intratumoral injection of the **OctaNPs** (0.1 mg/mL, 200 μl).

1.8 *In vivo* toxicity

OctaNPs (5 mg/kg) were administered via tail vein injection or intragastric administration into healthy BALB/c mice (five mice per group), respectively. PBS was used as a negative control. After seven days, the stomach and major organs (including heart, liver, kidneys, lungs and spleen) were harvested. Through a series of processes, including fixation in 10% neutral buffered formalin, embedding into paraffin and sectioning at 5 μm thickness, the tissues were stained with H&E and examined by means of a digital microscope (Olympus, CX31, Japan).

2. Additional Figures

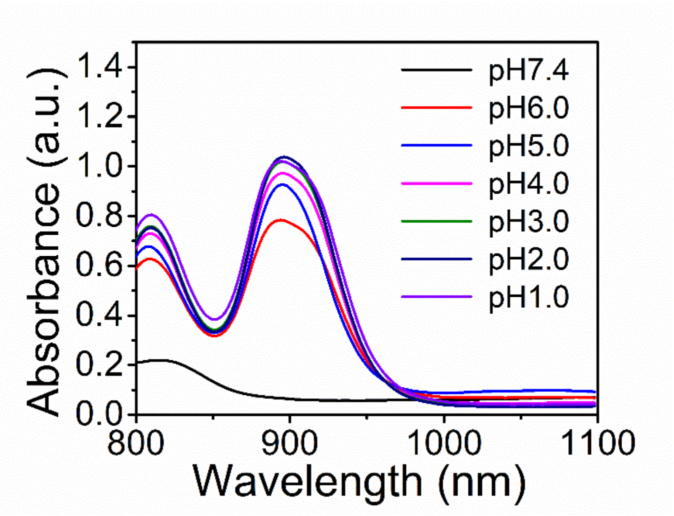

Figure S1 – Absorption of **1** at varying apparent pH values in THF.

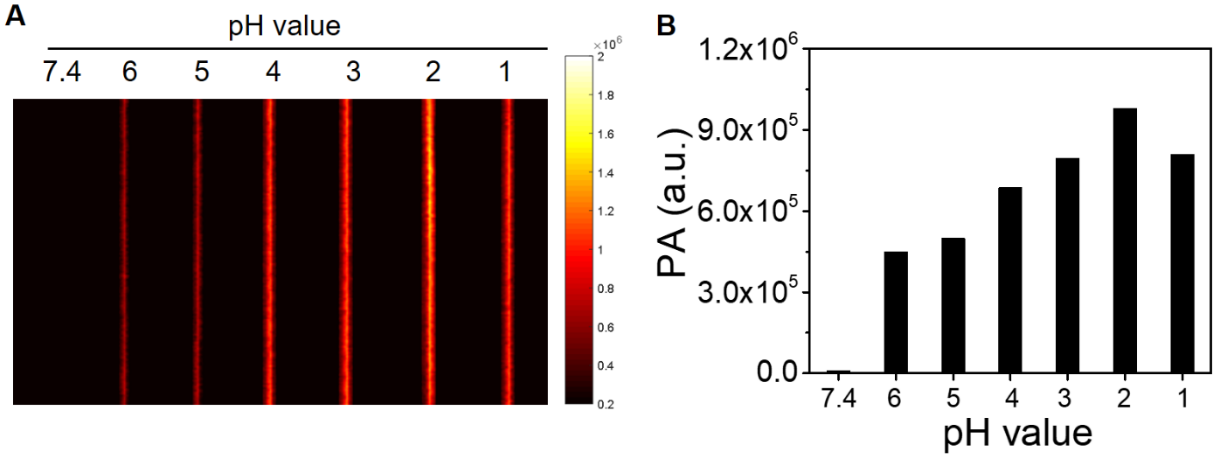

Figure S2 – (A) PA images of **1** (0.1 mg/ml) at varying apparent pH values in THF. (B) Quantified PA signals of **4** (0.1 mg/ml) at varying effective pH values in THF. Laser excitation was effected at 900 nm and the power was 15 mJ.

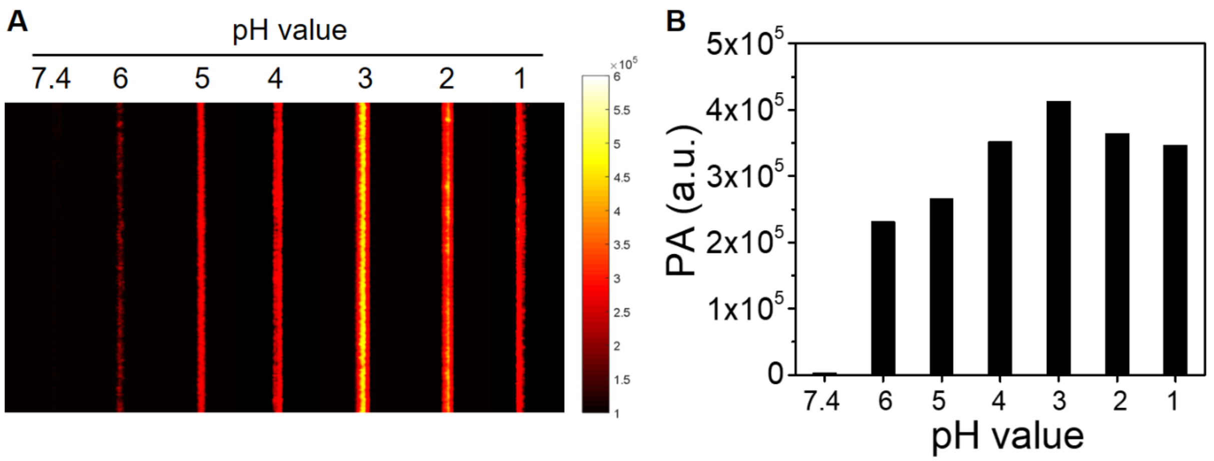

Figure S3 – (A) PA images of **4** (0.1 mg/ml) at varying apparent pH values in THF. (B) Quantified PA signals of **4** (0.1 mg/ml) at varying apparent pH values in THF. Laser excitation was effected at 1200 nm and the power was 15 mJ.

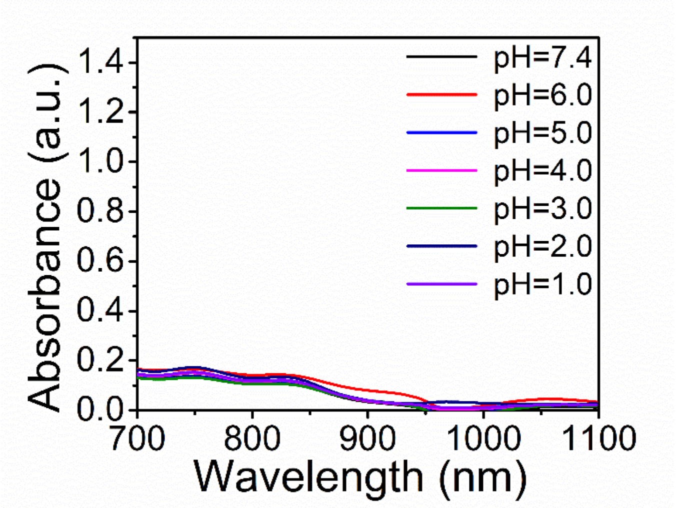

Figure S4 – Absorption of **NaphthNPs** (0.1 mg/ml) at varying pH values in deionized water.

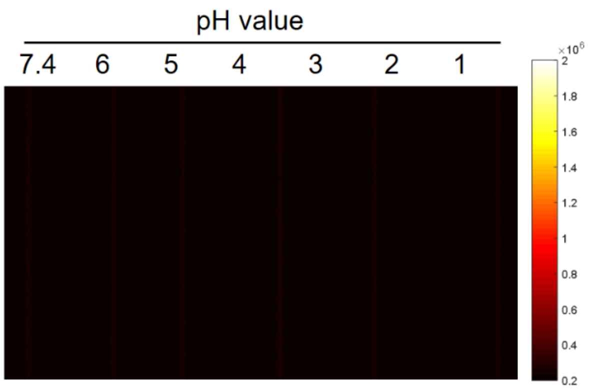

Figure S5 – PA images of **NaphthNPs** (0.1 mg/ml) at varying pH values in aqueous media. Laser excitation was effected at 900 nm and the power was 15 mJ.

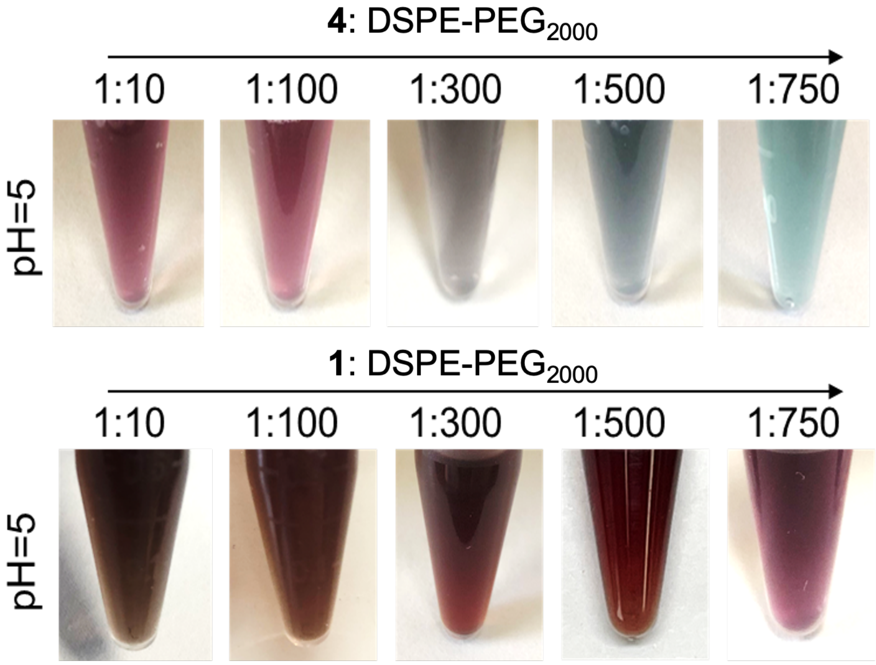

Figure S6 - Photographs of aqueous solutions of **OctaNPs** and **NaphthNPs** with different ratios of **1** or **4** and DSPE-PEG_{2000} at pH = 5.

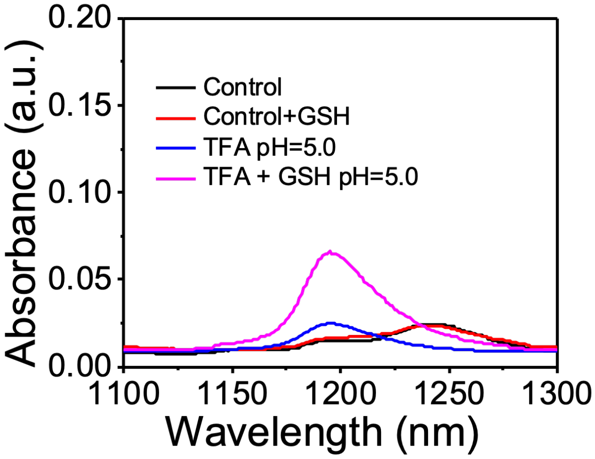

Figure S7 - Absorbance spectra of **OctaNPs** with GSH at pH = 7.4, TFA (trifluoroacetic acid) at pH = 5.0, TFA + GSH at pH = 5.0. GSH concentration = 40 μM

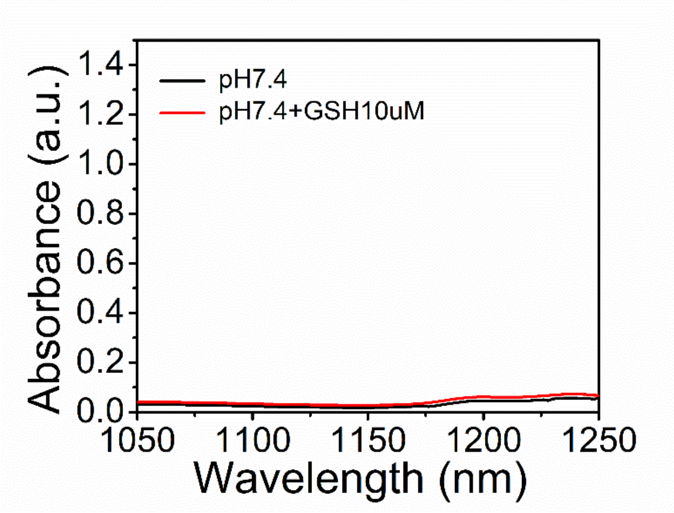

Figure S8 - Absorption of **OctaNPs** (0.1 mg/ml) with and without 10 mM GSH at pH 7.4 in an aqueous medium.

3. References

1. M. Ishida, S. J. Kim, C. Preihs, K. Ohkubo, J. M. Lim, B. S. Lee, J. S. Park, V. M. Lynch, V. V. Roznyatovskiy, T. Sarma, P. K. Panda, C. H. Lee, S. Fukuzumi, D. Kim and J. L. Sessler, *Nat. Chem.*, 2013, **5**, 15-20.

2. T. Sarma, G. Kim, S. Sen, W. Y. Cha, Z. Duan, M. D. Moore, V. M. Lynch, Z. Zhang, D. Kim and J. L. Sessler, *J. Am. Chem. Soc.*, 2018, **140**, 12111-12119.
